# Synthetic lethality between androgen receptor signalling and the PARP pathway in prostate cancer

**DOI:** 10.1038/s41467-017-00393-y

**Published:** 2017-08-29

**Authors:** Mohammad Asim, Firas Tarish, Heather I. Zecchini, Kumar Sanjiv, Eleni Gelali, Charles E. Massie, Ajoeb Baridi, Anne Y. Warren, Wanfeng Zhao, Christoph Ogris, Leigh-Anne McDuffus, Patrice Mascalchi, Greg Shaw, Harveer Dev, Karan Wadhwa, Paul Wijnhoven, Josep V. Forment, Scott R. Lyons, Andy G. Lynch, Cormac O’Neill, Vincent R. Zecchini, Paul S. Rennie, Aria Baniahmad, Simon Tavaré, Ian G. Mills, Yaron Galanty, Nicola Crosetto, Niklas Schultz, David Neal, Thomas Helleday

**Affiliations:** 1 Cancer Research UK Institute, University of Cambridge, Li Ka Shing Centre, Cambridge, CB2 0RE UK; 2 Department of Clinical and Experimental Medicine, University of Surrey, Guildford, GU2 7WG UK; 3 Science for Life Laboratory, Division of Translational Medicine and Chemical Biology, Department of Medical Biochemistry and Biophysics, Karolinska Institutet, S-171 21 Stockholm, Sweden; 4 Department of Urology, Central Hospital, 721 89 Västerås, Sweden; 5 Department of Pathology, Addenbrooke’s Cambridge University Hospital, Cambridge, CB2 0QQ UK; 6 The Wellcome Trust and Cancer Research UK Gurdon Institute, University of Cambridge, Cambridge, CB2 1QN UK; 7 The Vancouver Prostate Centre, Department of Urologic Sciences, University of British Columbia, Vancouver, BC Canada V6H 3Z6; 8 Institute of Human Genetics, Jena University Hospital, 07743 Jena, Germany; 9 Centre for Molecular Medicine Norway, Nordic European Molecular Biology Laboratory Partnership, University of Oslo, 0318 Oslo, Norway; 10 Prostate Cancer UK/Movember Centre of Excellence, Queen’s University, Belfast, BT9 7AE UK; 11 Nuffield Department of Surgery, University of Oxford, John Radcliffe Hospital, Headley Way, Headington, Oxford, OX3 9DU UK

## Abstract

Emerging data demonstrate homologous recombination (HR) defects in castration-resistant prostate cancers, rendering these tumours sensitive to PARP inhibition. Here we demonstrate a direct requirement for the androgen receptor (AR) to maintain HR gene expression and HR activity in prostate cancer. We show that PARP-mediated repair pathways are upregulated in prostate cancer following androgen-deprivation therapy (ADT). Furthermore, upregulation of PARP activity is essential for the survival of prostate cancer cells and we demonstrate a synthetic lethality between ADT and PARP inhibition in vivo. Our data suggest that ADT can functionally impair HR prior to the development of castration resistance and that, this potentially could be exploited therapeutically using PARP inhibitors in combination with androgen-deprivation therapy upfront in advanced or high-risk prostate cancer.

## Abstract (web summary)

Tumours with homologous recombination (HR) defects become sensitive to PARPi. Here, the authors show that androgen receptor (AR) regulates HR and AR inhibition activates the PARP pathway in vivo, thus inhibition of both AR and PARP is required for effective treatment of high risk prostate cancer.

## Introduction

Prostate cancer (PCa) is a major cause of male cancer-related mortality worldwide^1^. The androgen receptor (AR) is a ligand-inducible transcription factor that plays a key role in the initiation, growth and progression of PCa^2^. Therefore, androgen-deprivation therapy (ADT), which targets the androgen signalling axis, provides an effective first-line treatment for advanced PCa^3^. Progression to lethal castration-resistant prostate cancer (CRPC) is common and accompanied by restoration or maintenance of AR signalling, which is involved in the regulation of metabolism^4, 5^, cell cycle checkpoints^6^ and DNA repair^7–10^. The recent data also demonstrate mutations in *BRCA2, BRCA1*, and *ATM* genes in about 20% of advanced PCa^11^. PARP is a backup DNA repair pathway in cells that have lost *BRCA1, BRCA2* or *ATM* function^12^. As a result, *BRCA2*-deficient cells are acutely sensitive to PARP inhibition^13, 14^, a phenomenon known as synthetic lethality. In line with this, the emerging data demonstrate profound clinical responses using PARP inhibitors in CRPC mutated in *ATM, BRCA2* and *BRCA1* ^11, 15, 16^.

In PCa, a number of clinical studies have shown that ADT combined with radiotherapy, which induces DNA double-strand breaks (DSBs), is a more effective treatment option for locally advanced disease and is associated with better survival and disease-free outcome compared with radiotherapy alone^17, 18^. Previously, we and others have demonstrated that non-homologous end joining repair of DNA DSBs is affected by ADT, which could be one explanation for this increased sensitivity^7–10^. Since homologous recombination (HR) is also important in radiation-induced DSB repair, here we investigated a functional link between AR signalling and HR in PCa, which also could open up a novel therapeutic opportunity using PARP inhibitors.

In this study, we show that AR signalling regulates the HR and promotes MRN foci formation, leading to ATM activation in PCa. Our data suggest that the AR promotes the DNA damage response (DDR) by facilitating efficient accumulation of үH2AX and RAD51 foci, promoting HR. Blocking AR signalling in men receiving ADT activates PARP signalling and thereby inhibition of AR function is synthetically lethal with PARP inhibition in PCa.

## Results

### The AR promotes HR and DDR signalling

**Fig. 1:**
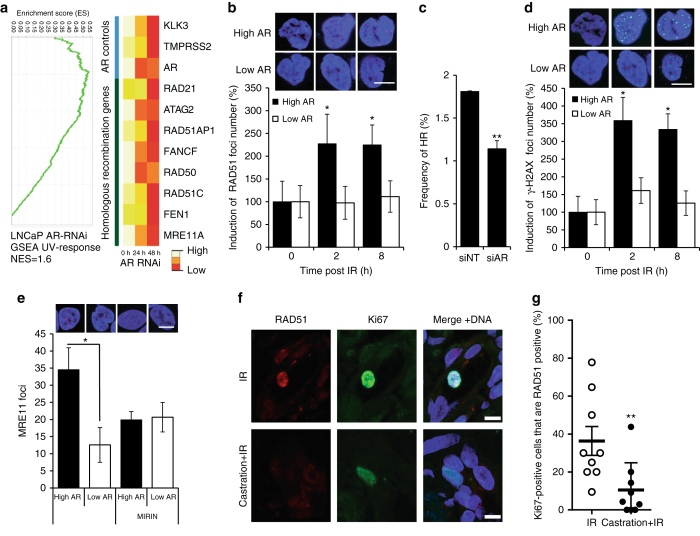
AR signalling regulates homologous recombination (HR) and the DNA damage response. **a** Heat map showing expression of homologous recombination regulators following AR RNAi knockdown, AR transcript and known AR targets/controls are shown mined from the microarray data from LNCaP cells^19^. **b** Time course of formation of RAD51 foci showing high content cytometry-based quantification of RAD51 nuclear foci number per nucleus in ‘low AR; and ‘high AR’ C4-2 cells in response to 10 Gy radiation. Statistical significance was determined using the Holm–Sidak method, with alpha = 5.000% (*n* = 3). *Scale bar* = 1 μm. **c** Quantification of gene conversion assay from analysis of GFP-positive C4-2-DRGFP cells transfected with non-targeting control siRNA (siNT) or siAR along with ISce1 endonuclease for 72 h followed by detection of GFP-positive cells by flow cytometry. *P*-value by two-sided Student’s t-test (*n* = 3); the data represent mean ± SEM. **d** Foci analysis time course showing high content cytometry-based quantification of the number of γH2AX foci in ‘low AR’ and ‘high AR’ C4-2 cell nuclei in response to radiation (10 Gy). Statistical significance determined using the Holm–Sidak method, with alpha = 5.000%. (*n* = 3). *Scale bar* = 1 μm. **e** High content cytometry analysis of MRN foci in ‘high AR’ and ‘low AR’ C4-2 cells treated with hydroxyurea (HU). *Upper panel* shows ‘high AR’ and ‘low AR’ nuclei with MRN foci (*red dots*), *lower panel* shows a histogram with means of the errors; *P*-value by two-sided Student’s *t*-test (*n* = 2). **f** Micrographs from biopsies showing examples of Ki67- and RAD51 positive cells and Ki67 positive alone. *Scale bar* = 5 μm. **g** Graph showing percentage of RAD51-positive cells in the population of Ki67-positive cells in biopsies after indicated treatment. *Error bars* shows SEM and ** = *P* < 0.01, Mann–Whitney test

Since AR has been shown to regulate DNA repair^7–10^, we carried out an in-depth analysis of the expression pattern of DNA repair genes induced by AR signalling and revealed a potential link between AR signalling and HR (Fig. 1a). In line with these results, we observed that the expression of RAD51, a key player in HR, was significantly upregulated in PCa compared with normal benign prostate tissue (Supplementary Fig. 1a). To interrogate the functional link between AR signalling and HR, we tested whether AR signalling affects ionising radiation (IR)-induced RAD51 foci formation. To test this, we used an isogenic CRPC model C4-2 cell line^19^, in which ‘high AR’ and corresponding ‘low AR’ levels are obtained by the doxycycline-mediated induction of a short-hairpin RNA targeting the AR (Supplementary Fig. 1b). In line with AR regulating HR genes, we observed decreased numbers of IR-induced RAD51 foci in ‘low AR’ cells (*P* < 0.05; 2- and 8 h post IR) in response to 10 Gy of radiation (Fig. 1b).

To test if there was a direct role of the AR in regulating HR, we performed a direct repeat-green fluorescent protein (DR-GFP)-based gene conversion assay in C4-2 cells with a stably integrated DR-GFP reporter. Consistent with its role in promoting RAD51 foci formation, cells with AR knockdown using a pre-validated siRNA showed a significant reduction of HR (*P* < 0.01) (Fig. 1c and Supplementary Fig. 1c), which appeared to be a true effect on HR, as no difference in cell cycle stage was observed (Supplementary Fig. 1d). Next, we determined in detail how the AR regulates the DDR and repair, and found a strong correlation between the DDR kinase ATM and the AR-regulated transcriptome of DNA repair genes in PCa cells (Supplementary Fig. 1e). Supporting this link, we observed an overall defect in IR-induced үH2AX foci formation in ‘low AR’ cells after 10 Gy of radiation (*P* < 0.05 2- and 8 h post IR) (Fig. 1d). In agreement with this, we found that men with PCa treated with degarelix, a gonadotropin-releasing hormone analogue, which blocks androgen signalling, had overall lower levels of үH2AX foci (Supplementary Fig. 2a). We observed an overall defect in IR-induced үH2AX foci formation in ‘low AR’ cells 2 h after 10 Gy of radiation in larger cell numbers by a high-content cytometry method (Supplementary Fig. 2b, c), suggesting that the AR influences the DDR directly. We also observed that siRNA depletion of ATM in ‘high AR’ cells reduces the үH2AX intensity comparable to ‘low AR’ levels (*P* < 0.01) (Supplementary Fig. 2d). A further link between the AR and ATM signalling was observed using an isogenic inducible AR point mutant (T878A) expressed in the PC3 cell line, in which doxycycline treatment triggered AR expression. We observed that from 48 h after exposure to ionising radiation, the ‘full AR’ cells grew faster than ‘AR null’ cells, but this differential response was eliminated by ATM inhibitor (KU55399) treatment (Supplementary Fig. 2e). No differential growth response was observed in AR null PC3-Empty Vector control cells (Supplementary Fig. 2f).

The ATM and other DDR proteins affect MRE11 activity to resect DNA ends, which is required for HR. Mining the AR transcriptome (GEO identifier GDS4113), we observed down-regulation of the MRE11 transcript by AR knockdown, which was also directly suppressed at the protein level by AR knockdown (Supplementary Fig. 2g, h). As ATM regulates MRE11 activity, we tested formation of hydroxyurea-induced MRE11 foci, which were higher in ‘high AR’ cells (*P* < 0.05) (Fig. 1e). That the MRE11 foci represent active MRE11 resection was demonstrated by addition of the MRE11 inhibitor Mirin, which reduced the number of MRE11 foci in “high AR” cells (Fig. 1e). Taken together, these data suggest that AR is required for effective ATM signalling in response DNA damage in PCa, influencing MRE11 mediated resection required for proficient HR.

To verify these findings in patients, a prospective study was set up with PCa patients. PCa was diagnosed at biopsy and patients then underwent pharmacological castration with neo-adjuvant leuprolide. A second biopsy was taken 8 weeks post leuprolide treatment. Half the cohort had leuprolide before radiation treatment, the other half started radiotherapy before leuprolide. The proportion of cell positive for Ki67 and RAD51 were quantified using immune-fluorescence in a randomly selected sub cohort of 11 patients treated with ADT before 2 × 5 Gy fractionated radiation and from 12 patients treated with fractionated radiation alone (Fig. 1f). In all biopsies, the intensity of Ki67 and RAD51 in the nucleus for all epithelial cells was measured. The fraction of RAD51-positive cells in the population of cycling cells (Ki67 positive) was calculated for each biopsy. A significantly lower (*P* < 0.01) fraction of RAD51-positive cells in the population of cycling cells was seen in tissue from those patients who received ADT before radiation compared with those treated with radiation alone, suggesting that, as in PCa cell lines, ADT treatment also hampers HR repair after radiation in patients (Fig. 1g).

### Androgen deprivation therapy ﻿activates PARP in PCa

**Fig. 2:**
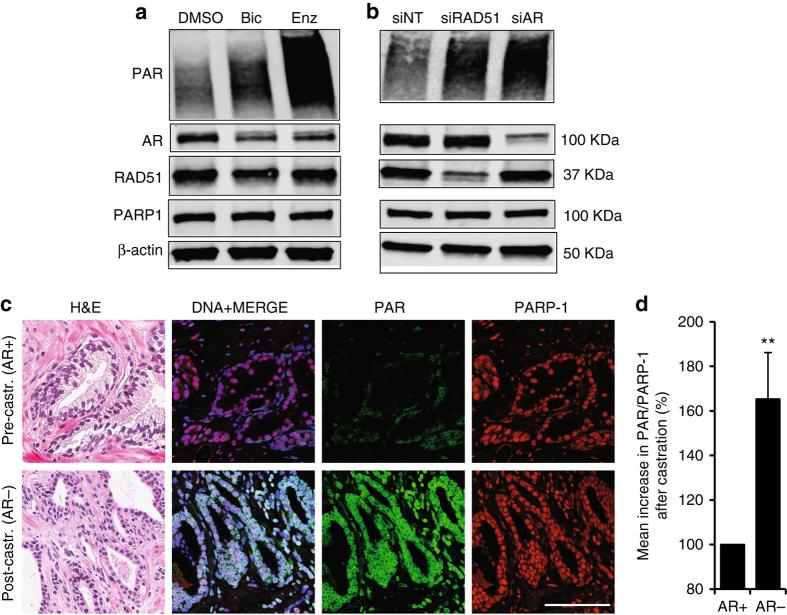
Androgen deprivation therapy triggers PARP activation. **a** Western blot showing C4-2 cells treated for 72 h with indicated agents (10 µM). **b** Western blot showing C4-2 cells transfected with indicated siRNA (20 nM). **c** Immunofluorescence microscopy images showing relative intensities of PAR Poly(ADP Ribose) and PARP1 (Poly ADP-ribose polymerase 1) in PCa tissue pre- (pre-castration or AR+) and post leuprolide (post-castration or AR-) treatment (8 weeks). *Scale bar* = 100 μm. **d** Histogram shows PARP activity in PCa patients pre- and post-leuprolide treatment (One sample t-test, two-tailed, test value 100)

Previously, we demonstrated that PARP1 is activated in HR-defective cells, and that sensitivity to PARP inhibitors is related to PARP activation^20^. Recently, it was demonstrated that AR upregulation can cause redioresistance^21^. Since we found that the AR promotes HR, we hypothesized that AR inhibition by ADT may impair HR, leading to an increase in backup PARP activity. To test this hypothesis, we treated C4-2 cells with the non-steroidal antiandrogens bicalutamide (Bic) and enzalutamide (Enz). Both drugs led to an increase of PARP activity (Fig. 2a; Supplementary Fig. 7a). To further investigate links between ADT, HR and PARP activity, we transfected C4-2 cells with siRNA against RAD51 and AR. We observed an increase in PARP activity in both the RAD51- and AR-depleted cells, suggesting that ADT impairs HR, leading to an increase in PARP activity (Fig. 2b; Supplementary Fig. 7b). We next tested whether the increase in PARP activity was due to AR inhibition induced unresolved DNA damage. IR-induced DSBs were directly measured in ‘high AR’ and ‘low AR’ C4-2 cells using neutral comet assay but we did not find any difference in DSBs in ‘high AR’ or ‘low AR’ context, indicating that the AR inhibition-induced PARP activation was not due to increased DSBs in the DNA (Supplementary Fig. 2i).

To test if such signalling also occurs in patients, we used the prospective study with PCa patients described above. Immunofluorescence signalling levels of PARP1 and its substrate PAR were quantified in cancer areas in corresponding paired slides (Fig. 2c and Supplementary Fig. 3a). To measure the activity of the PARP1 enzyme, we calculated the ratio of PAR to PARP1 intensity in the nuclei. In leuprolide-treated patients, we found a significant increase in PARP1 activity as reflected in increased PARylation (*P* = 0.003) compared with biopsies taken from the same patients before castration (Fig. 2c, d), suggesting that PARP1 is activated as a result of androgen deprivation, further strengthening our findings showing that AR regulates HR in PCa.

It could be speculated that not only the activity of PARP, but also the PARP1 gene expression is higher as a consequence of the HR defect. Since our biopsies have part cancer and part non-malignant tissue, we quantified PARP1 mRNA using RNA-fluorescence in situ hybridisation (RNA-FISH) while maintaining spatial resolution in order to be able to correlate PARP mRNA to the pathologically verified cancer areas. We found no meaningful increase of PARP mRNA density levels in cancer tissue, as was also the case of PARP protein levels (Supplementary Fig. 4). These data are in line with previous demonstration that PARP is activated at stalled replication forks caused by the HR defect^22^ thus ruling out that a HR defect would affect PARP1 gene expression directly.

### Synthetic lethality between the AR and poly (ADP-ribose) polymerase

Previously, we demonstrated that HR defective cells show increased reliance on PARP activity and demonstrated a synthetic lethality between PARP- and *BRCA*-mutated cancers^13, 14^. Here we observed an ADT-induced HR defect, suggesting that it may be possible to generate a context dependent synthetic lethality with PARP inhibitors following ADT. Because we observed high PARP activity after ADT in patients (Fig. 2) and also high PARP activity in PCa cells (Supplementary Fig. 3b), we reasoned that a combined inhibition of both pathways may selectively induce a synthetic lethality in PCa.

**Fig. 3:**
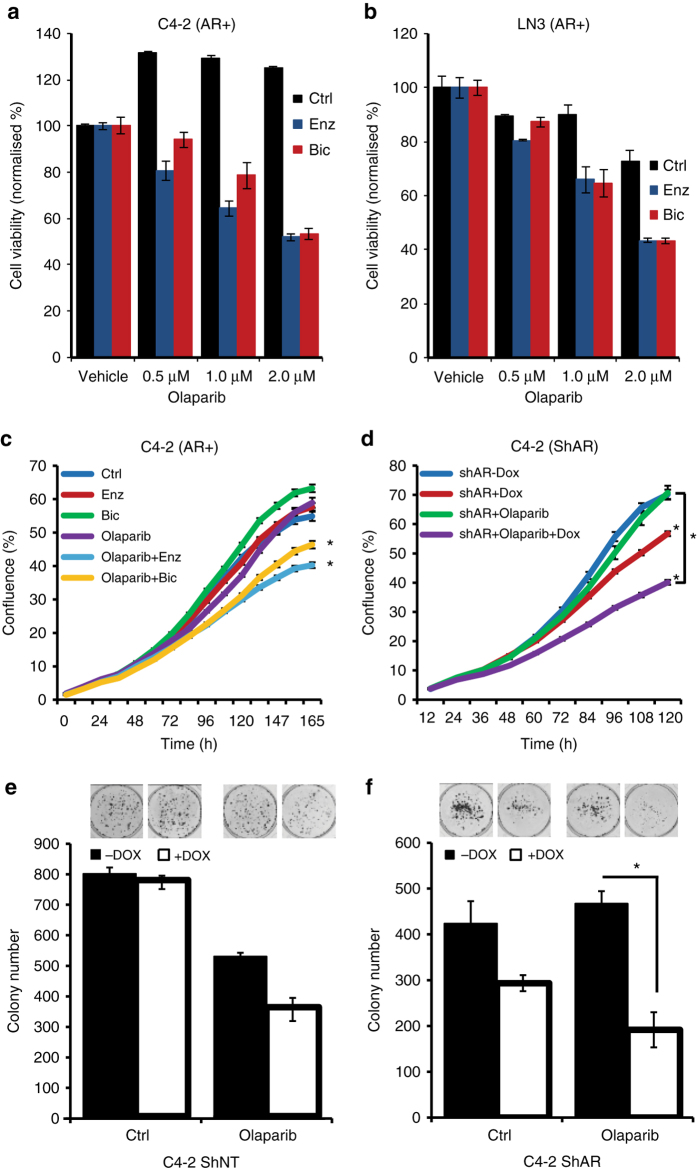
Synthetic lethality between AR and PARP pathways in PCa. **a**, **b** Viable fraction assessed by MTS assay, of C4-2 (**a**) and (**b**) LN3 cells treated with the indicated doses of Olaparib and/or enzalutamide or bicalutamide (10 μM) for 7 days or until 95% confluence was reached; *P*-value by two-sided Student’s *t*-test; *bars* show mean ± SEM. **c** Live cell imaging confluence analysis (Incucyte) of C4-2 cells treated with Olaparib (1 μM), enzalutamide (10 μM), bicalutamide (10 μM) or combined treatment, statistical significance calculated by two-way ANOVA. **d** Live cell imaging confluence analysis (Incucyte) of ‘high AR’ and ‘low AR’ C4-2 cells treated with doxycycline and/or Olaparib (1 μM), statistical significance calculated using two-way ANOVA. **e**, **f** Clonogenic survival assay for C4-2 cells with either inducible shNT (**e**) or shAR (**f**); cells were treated with doxycycline and 1 μM Olaparib, *P*-value by two-sided Student’s *t*-test; *bars* show mean ± SEM. All experiments were independently performed in triplicates. The data represent means ± SEM. *P*-values from significant two-sided Student’s *t*-tests are given (* = *P* < 0.05)

To test this hypothesis, we used the anti-androgens bicalutamide or enzalutamide in combination with PARP inhibitor Olaparib. This decreased cell viability of the AR-positive cell lines C4-2 and LN3 (Fig. 3a, b), which was also confirmed by using other PCa cell lines (Supplementary Fig. 5a–d). The proliferation of C4-2 cells was also reduced by co-treatment with Olaparib and either bicalutamide or enzalutamide, (Fig. 3c). Also, AR shRNA-mediated knockdown resulted in decreased proliferation, and this was further decreased by Olaparib (Fig. 3d). Consistent with that finding, the synthetic lethality was AR dependent, knockdown of the ectopically expressed T878A *AR* mutant in PC3-T878A and C4-2 cell lines (which endogenously express this mutant) had only marginal effects on cell viability (Supplementary Fig. 5f, g, 5e, f). The clonogenic potential of C4-2 cells expressing the control shNT was unchanged by doxycycline treatment, while C4-2 cells expressing control shNT cells treated with Olaparib formed fewer colonies independent of doxycycline treatment (Fig. 3e). In contrast, the clonogenic potential of doxycycline-treated C4-2 cells expressing shAR ‘low AR’ was severely compromised by Olaparib (*p* < 0.05) (Fig. 3f), indicating synthetic lethality.

Two primary modes by which PARP inhibitors act are as follows: (A) catalytic inhibition of the PARP enzyme and consequently blocking PARylation of proteins and (B) trapping of PARP on ssDNA intermediates generated by the DNA damage^23, 24^. If left unrepaired, these PARP trapped-ssDNA intermediates may be converted into DSBs in S-phase and may lead to cell death. In our study, we observed loss of the growth inhibitory effect of Olaparib in C4-2 cells in which PARP1 was knocked down with RNAi, indicating that the growth inhibitory effect of Olaparib could be partially manifested via the trapping of PARP1 (Supplementary Fig. 5g). These experiments suggest that AR and PARP may act through functionally distinct pathways that converge to promote cell growth under genotoxic stress, further strengthening the model of contextual synthetic lethality.

**Fig. 4:**
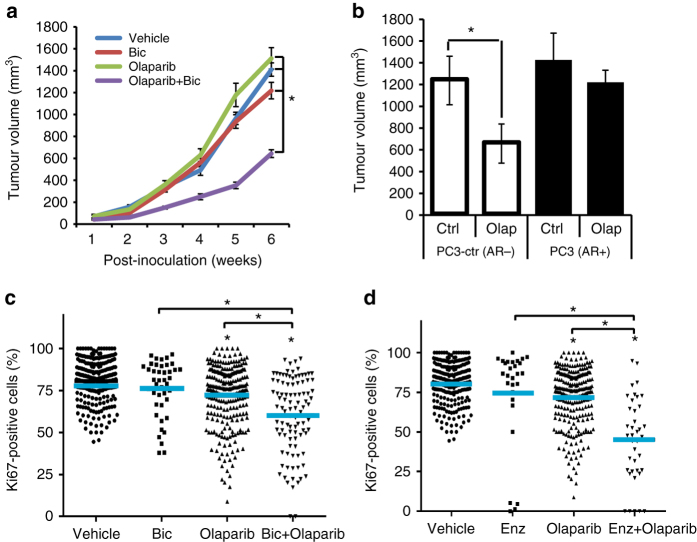
Dual inhibition of AR and PARP1/2 function represses PCa growth. **a** Tumour xenografts of C4-2 cells. NSG mice were treated with DMSO (vehicle) or bicalutamide and/or Olaparib for 6 weeks, statistical significance calculated using two-way ANOVA. **b** Tumour xenografts of PC3-ctl or PC3-AR cells, NSG mice were administered with DMSO (vehicle) or Olaparib as indicated, *P*-value by two-sided Student’s *t*-test. **c**, **d** Quantification of ki67 expression in ex vivo culture of human PCa treated with (**c**) bicalutamide (10 μM) or (**d**) Enzalutamide (10 μM) and/or Olaparib (2 μM) for 72 h, significance calculated by Mann–Whitney U test

Next, we wanted to evaluate the synthetic lethality between ADT and PARP inhibitors in vivo, and therefore generated tumour xenografts of C4-2 cells. In the vehicle, bicalutamide- and Olaparib-treated groups a progressive increase in tumour volume was observed (Fig. 4a). However, combined treatment with bicalutamide and Olaparib significantly suppressed the growth of the xenografts (*P* < 0.05) and decreased tumour volume (Fig. 4a and Supplementary Fig. 6a) without any obvious toxicity to mice (Supplementary Fig. 6b). To extend these findings to a wider context, we generated xenografts of AR null PC3-ctr or PC3-AR-expressing cells (Supplementary Fig. 6c) and administered vehicle or Olaparib to the mice, demonstrating decreased tumour volume with Olaparib only in the AR null context in PC3-ctr xenografts (Fig. 4b and Supplementary Fig. 6d, e) without any toxicity indications on mice (Supplementary Fig. 6f). To test this in clinically relevant material, we used an ex vivo human tumour culture assay, where treatment of PCa tissue explants with bicalutamide or enzalutamide had no effect on proliferation index (measured by Ki67), but proliferation was strongly repressed by the combination of Olaparib with bicalutamide or enzalutamide (*P* < 0.05) (Fig. 4c, d). Treatment with Olaparib alone and by combined bicalutamide and Olaparib increased levels of cleaved caspase-3 (CC3) (a marker of apoptosis) compared to controls or single treatments (Supplementary Fig. 6g). However, the combination of enzalutamide and Olaparib did not result in increased CC3 (Supplementary Fig. 6h).

## Discussion

Radiotherapy combined with ADT is now standard radical management for men with intermediate- and high-risk PCa. ADT alone is used for palliative treatment in advanced PCa and relapse is common. Previous reports of altered gene expression of DNA repair genes in CRPC^25^, have now been extended with identification of somatic mutations in DNA repair genes in about 20% of CRPC^11^, particularly in BRCA2 and ATM. The likely evolutionary advantage of mutations or loss of *BRCA2* and *ATM* in CRPC is that they act as tumour suppressors in the CRPC. Remarkably, our finding that inhibition of AR signalling results in reduced HR and ATM signalling suggests that HR is impaired in the early phases of ADT treatment. It is tempting to speculate that HR or ATM signalling would have a tumour suppressor function after ADT and that down-regulation of HR is a mechanism whereby the tumour increases its ability to survive treatment.

Mechanistically, we have demonstrated that loss of the AR leads to downregulated HR gene expression, reduced ATM signalling and MRE11 foci formation. MRE11 activity is required for resection at DSBs to generate a substrate onto which RAD51 can be loaded. Hence, our data suggest that HR is impaired by ADT at the stage of DNA double-strand end processing, which is unsurprising, since expression of many HR genes is downregulated. It is well established that PARP is a backup repair system required for survival in absence of HR^13, 14^. Here we demonstrate, both in cell lines in vitro and in a prospective clinical study, that PARP activity is increased in PCa tissue following ADT, in line with the hypothesis that HR is downregulated after ADT in vitro.

Different PARP inhibitors have different trapping abilities^24^, and hence it is important to determine whether PARP trapping is important. Our finding that toxicity is removed by PARP1 siRNA suggests that PARP trapping is relevant for the manifestation of toxicity, which is in line with current perception of the mechanism of action for PARP inhibitors in killing HR defective cells^24^.

Recent reports indicate remarkable responses to PARP inhibitors in the HR defective cohort of CRPC^15, 16^. About 20% of CRPC have mutations in either *BRCA2* or *ATM* ^11^, and an additional ~ 15 % have mutations in other HR genes. The remaining two-third of CRPC likely gained ADT resistance through means other than mutation of HR genes, probably explaining why they do not respond to PARP inhibitor therapy^16^. Hence, mutational loss of HR is a possible way of generating ADT resistance and development of CRPC. Here, we find an immediate downregulation of HR in response to ADT, which is a likely reflection of these cells acutely improving fitness for survival. ADT is commonly used in early treatment of advanced PCa, but this is not a curative treatment. Here, we demonstrate that combining ADT with PARP inhibitors improves ADT and may increase the possibility of receiving more profound and long-lasting responses.

We suggest that the ADT and PARP inhibitor combination treatment is likely to be most effective in treatment of the early disease, rather than at the late CRPC stage. The HR mutated CRPC would not benefit as they already are defective in HR and would respond to PARP inhibitors in monotherapy, as already demonstrated^16^. CRPC that acquired resistance by means other than mutating HR genes would not benefit either as they are likely not responding to ADT at all, and hence would not downregulate HR genes and would not acquire PARP inhibitor sensitivity.

PARP inhibitors are generally well tolerated in patients and in combination with ADT, the HR defect would be predominantly restricted to the prostate tissue and toxicity would only occur there. Hence, we argue that ADT and PARP inhibitor combination treatment may be particularly beneficial in newly diagnosed high-risk PCa as (1) they have not generated castration resistance by means other than loss of HR and (2) the synthetic lethality is confined to the prostate and is likely to be well tolerated. The ADT and PARP combination treatment may be offered not only to newly diagnosed metastatic PCa but potentially as adjuvant treatment in high-risk men undergoing prostatectomy or radiotherapy.

## Methods

### Reagents/antibodies/consumables

Methyltrienolone (R1881) and enzalutamide were obtained from Perkin Elmer and Axon Medchem respectively. Camptothecin, dihydrotestosterone (DHT), DMSO, doxycycline, bicalutamide, hydroxyl urea and Mirin were obtained from Sigma. Olaparib was purchased from LC laboratories and ATM inhibitor KU0055399 from Calbiochem. Cell culture media, foetal bovine serum (FBS) and other cell culture reagents including antibiotics were obtained from Life Technologies. Antibodies were obtained from commercial suppliers as below and used at recommended dilutions for each application:

1. Phospho-Histone H2A.X (Ser139) (JBW301; cat # 05-636) from Millipore—used in foci analysis, үH2A.X (phosphoS139; cat # ab2893) from Abcam used for immuno histochemistry.
2. Rad51 (cat no. H-92; sc8349) from Santa Cruz Biotechnology.
3. Mre11 (cat no. 4895 S) from Cell Signaling Technology.
4. PAR antibody (cat no. GTX75054 from Source Bioscience).
5. AR (AR441; cat no. M356201-2 from Dako).
6. Beta-actin antibody (Rabbit actin: Cell Signaling Technology (cat no. 4970).
7. Mouse actin: Abcam (cat no. ab6276), 1:5000).
8. IgG-Alexa 555 from Life Technologies.
9. PARP1, H-250 Cat. no. sc-7150 from Santa Cruz Biotechnology.

### Cell culture

Unless stated otherwise, all cell lines were verified by genetic profiling of polymorphic short tandem repeat (STR) loci as per ATCC standards. We used AmpFISTR test or GenePrint10 test (Promega, Madison, WI) and analysed all the data using GeneMapper v4.0 software. LNCaP, C4-2, PC3, PNT1a, and DUCaP cells were obtained from commercial suppliers and grown in RPMI cell culture medium containing 10% FBS and 1% Penicillin/Streptomycin in a humidified incubator at 37 °C with 5% CO_2_. We generated and cultured C4-2-NT control (expressing non-targeting RNA; siNT) and C4-2-shAR cells as described earlier^19^. LNCaP-LN3 cells have been described previously^26^.

### Analysis of γH2AX and Rad51 foci in cells

C4-2 ‘high AR’ and ‘low AR’ shRNA cells were grown in RPMI medium containing 10% hormone-depleted serum and cells were treated with R1881 (1 nM) for ‘high AR’ or doxycycline (1 µg/ml) for 72 h to induce shRNA expression for ‘low AR’. Cells were exposed to IR (10 Gy) using a Cs137 source. For imaging, cells were grown in Ibidi ibiTreat 8-well µ-slides (Ibidi GmbH, Germany), pre-cleared with PBS containing 0.2% Triton-X-100 for 1 min then fixed in 3% PFA-2% sucrose solution in PBS for 10 min, washed 3× with PBS and blocked in Odyssey buffer for 30 min at room temperature. Cells were incubated with the combination of antibodies against үH2AX (1:2500) and Rad51 (1:500) overnight in Odyssey blocking solution and washed 3× with PBS containing 0.1% Triton-X-100. Cells were incubated with secondary antibodies Alexa Fluor^®^ 488 and Alexa Fluor^®^ 647 antibodies (both 1:1000; Life Technologies) for 1 h at room temperature. Cells were again washed with PBS containing 0.1% triton-X-100 and DNA counterstained with DAPI for 10 min followed by mounting with vectashield (Ibidi). Images were acquired on a Leica SP5 confocal microscope (Leica Microsystems Ltd). Three channels were used for DAPI (blue), үH2AX_AlexaFluor488 (green) and Rad51_AlexaFluor647 (far red). For analysis, the Leica image file was imported directly into Columbus software (PerkinElmer, UK). Images were analysed using a custom analysis protocol set up on the three channels. The DAPI channel was used to segment individual nuclei using standard algorithms within the software. үH2AX and Rad51 signal per nucleus was calculated by the quantification of the green and far red signals within the DAPI nuclear mask. The ‘find spots’ function was added into the protocol to detect үH2AX foci (green channel) and Rad51 foci (far red channel) within the nucleus, using appropriate settings to detect each individual focus. Functions were then added in to calculate morphology allowing foci number to be extracted.

### Immunohistochemistry, image processing, nuclei segmentation and spot quantification for γH2AX

Immunohistochemistry was performed on 3 μm sections from formalin-fixed, paraffin-embedded samples using an automated immunostainer with cover tile technology (Bond-III system, Leica Biosystems). A commercial antibody to үH2AX (1:400 dilution) was used as the primary antibody. Antigen retrieval was carried out using the combination of heat and Bond Epitope Retrieval Solution 1 (Leica Biosystems). The Bond™ Polymer Refine Detection kit (Leica Biosystems) was used for visualising the antigens. Negative control experiments, in which primary antibodies were omitted, resulted in a complete absence of staining.

All the images were processed with FIJI software (or ImageJ, National Institute of Health, Bethesda, MD) using a set of semi- and fully-automated custom-made macros. Briefly, artefacts within the image, i.e., bubbles, dust or out-of-focus regions were detected and removed upon user validation. Haematoxylin and diaminobenzidine (DAB) staining were then automatically separated as individual channels using a macro calling the ‘colour deconvolution’ plugin^27^ set with the “H&E DAB” vectors. A final macro was used to discard any secretion feature that could be further detected as a positive signal within the DAB channel. Processed DAB images were then analyzed with Columbus software (PerkinElmer, Waltham, MA). Nuclear segmentation was performed and small, large or elongated objects were removed. However, there was a mixed population of cancer and sparse stromal cells; a novel method was designed to select cells clustered in a cancer area automatically. Briefly, regions were defined as an extension by 50 pixels (12.5 μm) around each nucleus. By default, as no overlap is allowed by Columbus, measured areas for clustered cells were smaller than isolated stromal cells. Spot detection was finally achieved only for individually sorted cancer cells. Low-amplitude (maximum over local background intensity) spots were discarded to limit the contribution of false positives.

### Plasmids, siRNA and transient transfections

In order to achieve potent reduction in target mRNA expression, siRNA smart pool was used for gene knockdown (Dharmacon/Life Technologies). Transient transfections with siRNA were performed using Lipofectamine RNAiMAX transfection reagent (Life Technologies) as per manufacturer’s recommendations. We performed reverse transfections and used 25 nM siRNA in all knockdown experiments; MMTV-Luc and Renilla-Luc reporter plasmids were transfected using Lipofectamine 2000 (Life Technologies) as per manufacturers’ instructions.

### DR-GFP assay

C4-2 cells containing the DR-GFP reporter were generated by transfecting the cells with Lipofectamine 2000 (Thermo Fisher Scientific) and stable clones (designated C4-2 DRGFP cells) were selected on puromycin (2 µg/ml) and maintained in RPMI with 10% FBS. These cells were seeded in six-well culture dishes and transfected using Lipofectamine 2000 reagent with ISce1 (0.75 µg per well) and siRNA (50 nM). After 72 h, cells were collected and the percentage of GFP-positive cells was determined by flow cytometry using BD FacsCaliber (BD Biosciences). The data analysis was carried out using Flowjo software (Tree Star Inc.).

### Western blot analysis

Samples subjected to SDS-PAGE were transferred to a PVDF membrane (Amersham) and transfer efficiency was checked with Ponceau red. The membrane was blocked with Odyssey blocking buffer for 1 h at room temperature and washed with TBS-T (TBS, 0.05% Tween-20), then incubated with the indicated primary antibody dilutions as per manufacturer’s instruction overnight at 4 °C. Upon washing with TBS-T, membrane was incubated with HRP antibody for 1 h at room temperature and chemiluminiscence detected by Licor Odyssey instrument.

### Leuprolide clinical study design

After ethical approval from the regional ethics committee of Uppsala University (EPN Dnr 2011:066), patients with localised, i.e., non-metastatic PCa, eligible for curative RT, were enrolled in the study. After the completion of a written informed consent, the patients were allocated to one of the two study arms.

In Arm 1, 25 patients received neo-adjuvant pharmacological castration with leuprolide, a GnRH analogue, followed by external beam radiotherapy in daily 2 Gy fractions to a total dose of 78 Gy. In Arm 2, 23 patients first received radiotherapy, in 2 Gy daily fractions for 5 consecutive days, followed by neo-adjuvant leuprolide and then an equivalent, higher RT dose to a total of 82 Gy. Before treatment, needle-core biopsy specimens were obtained from all patients. In Arm 1, a second biopsy was taken 8 weeks after the leuprolide injection, i.e., before radiotherapy was started and a third biopsy about 3 h after the fifth fraction. In Arm 2, a second biopsy was taken about 3 h after the fifth radiation fraction, i.e., before hormone treatment was initiated and a third biopsy 8 weeks after the administration of leuprolide.

### Histology and immunofluorescence from the Leuprolide clinical study

Paraffin embedded prostate needle biopsies were sectioned and stained with H&E. In all biopsies, cancer areas were assessed according to the Gleason system and annotated by an uropathologist. Two biopsies, predominantly cancer, were picked from each biopsy session to be further sectioned for immunofluorescence analysis. After deparaffinisation and rehydration of the slides, antigen retrieval was performed with R-Buffer A (Electron Microscopy Sciences) in a pressure cooker. Blocking was performed with 2% bovine serum albumin for 1 h and the sections were subsequently incubated with different primary antibodies at 4 °C overnight. Rinsing was performed 3 ×5 min in TBS buffer before incubation with the secondary antibodies for 1 h at room temperature. After counterstaining of DNA, slides were mounted with ProLong Gold (Molecular Probes).

For PARP-1 and PAR measurement, slides were stained with the primary antibodies PARP-1 (1:500, H-250, Santa Cruz Biotechnology) and PAR (1:500, H10, Santa Cruz Biotechnology) and the secondary antibodies donkey IgG–Alexa Fluor 488 (1:500; Molecular Probes) and donkey anti-rabbit IgG–Alexa Fluor 555 (1:500; Molecular Probes). DNA was counterstained with TO-PRO-3 iodide (2 μM, Molecular Probes). Images from a cancer area with a good immunofluorescence signal were selected from each biopsy. The corresponding areas in the H&E-stained section were identified for histological verification of the cancer area. Two areas from each slide containing 300–600 cells were chosen for analyses. TO-PRO-3 was used as a DNA marker. All images were analysed, with respect to average staining intensity inside the nuclei and in the cytoplasm. The nuclear area was defined by the TO-PRO-3 signal. All measurements were performed using a program written in-house for National Institutes of Health (NIH) ImageJ. Fluorescence images were obtained with a Zeiss LSM 780 inverted confocal microscope, using a Plan-Apochromat 40×/NA 1.2 objective. Through-focus maximum projection images were acquired from optical sections 0.5 μm apart and with a section thickness of 1.0 μm. H&E-stained images were obtained with a Leica scan system.

For RAD51 and Ki67 measurement, slides were stained with the primary antibodies RAD51 (1:400, ab63801, Abcam) and Ki67 (1:250, MIB-1, Dako) and the secondary antibodies were donkey anti-mouse IgG–Alexa Fluor 564 (1:500; Molecular Probes) and donkey anti-rabbit IgG–Alexa Fluor 647 (1:500; Molecular Probes). DNA was counterstained with DAPI (1 μg/ml, Thermo Scientific). Tiled images covering the whole biopsy were acquired for each biopsy with a Zeiss LSM 780 inverted confocal microscope, using a Plan-Apochromat 20×/NA 0.8 objective. Tiled images were created from single images with a section thickness of 4.0 μm. Measurements of the average intensity of Ki67 and RAD51 in nucleus of epithelial cells were done for all biopsies with an in-house written program for National Institutes of Health (NIH) ImageJ. Cells were considered Ki67 positive if their average nuclear fluorescence for Ki67 was five times higher than the average Ki67 fluorescence for all nuclei, or if the five times average Ki67 fluorescence was above 200 in a scale from 0 to 255. Ki67-positive cells were considered RAD51 positive if the RAD51 average intensity in the nuclei was two times higher than the average RAD51 intensity for all nuclei. For each biopsy the percentage of Ki67-positive cells that were RAD51 positive was calculated. Biopsies with fewer than five Ki67-positive cells were excluded from the analyses.

### Degarelix clinical study design

Full ethical approval was obtained (11/H0311/2) for clinical studies NCT01852864 and NCT00967889. Written informed consent was received from participants prior to inclusion in the study which was carried out at Addenbrooke’s hospital in Cambridge. A total of 27 patients with high-risk organ-confined PCa were treated with 240 mg of degarelix s.c. 7 days before surgery. Fresh PCa samples were obtained at the time of radical prostatectomy and snap frozen. Confirmation of castrate levels of plasma testosterone in degarelix-treated patients was achieved by mass spectrometry. These results were compared with matched controlled samples from 20 untreated patients. Tissues were spotted on microscopic slides to generate tissue microarrays.

### C4-2 PARP activity assay

C4-2 cells were transfected with 20 nM of siRNA of RAD51 (Invitrogen), AR (Gift from Oxford) or AllStars Negative control (Qiagen) using Interferin (Polyplus). siRNA and interferin mixture were removed after 24 h and replaced with fresh media or cells were treated with 10 µM of Bicalutamide and Enzalutamide for 72 h. At the end of incubation, cells were washed twice in PBS and scraped. Later lysis buffer (50 mM Tris, pH 7.4, 250 mM NaCl, 5 mM EDTA, 1% Triton X-100, Roche protease inhibitor 10×, Thermo phosphatase inhibitors 100×) was added to the cell pellet, which was then and kept on ice for 30 min followed by sonication for complete lysis. The protein concentration was measured and proteins were separated on a 4–12% Bis-Tris acrylamide gel followed by transfer to a nitro cellulose membrane. The membrane was blocked in odyssey blocking buffer followed by overnight incubation with primary antibody (pADPr (Santa Cruz, clone10H), AR (Santa Cruz, N-20), PARP1(Santa Cruz, H250), RAD51 (Santa Cruz, H92), β−actin (Abcam, ab49900) and 1 h incubation with fluorescent secondary antibodies (HRP-conjugated rabbit and mouse antibodies from LICOR). Odyssey LICOR was used to analyze western blot band.

### Histological and immunofluorescence evaluation of PAR and PARP-1

All prostatic needle biopsy specimens were embedded in paraffin, sectioned and stained with haematoxylin and eosin (H&E). In all specimens, the cancer area was assessed according to the Gleason system^28^ and marked by a uro-pathologist. Two cancer-rich specimens from each batch of biopsies were further sectioned for immunofluorescence analysis. These sections were deparaffinised and rehydrated before antigen retrieval with Tris/EDTA (Citrus buffer, pH 9) in a pressure cooker. After blocking with 2% BSA, the sections were incubated with different primary antibodies at 4 °C overnight. After extensive rinsing, the sections were incubated with the secondary antibodies (donkey anti mouse IgG-alexa 488 (1:500), Molecular probe and donkey anti rabbit IgG-alexa 555 (1:500), Molecular probe) for 1 h at room temperature. DNA was counterstained with TO-PRO-3 iodide (Molecular probe) and slides mounted with pro long gold (Molecular probe). Dual staining was performed on the same slide PARP1 (1:200, H-250, sc-7150, Santa Cruz) together with PAR (1:200, pADPr (10H): sc-56198, Santa Cruz Biotechnology). Images from a tumour area with a good degree of immunofluorescence signals were selected from each biopsy. The corresponding areas in the HE-stained section were identified for histological verification of the tumour area. Selected areas from each slide containing 300–600 cells were chosen for analyses. TO-PRO-3 was used as a DNA marker. All images were analysed, with respect to medium intensity inside the nuclei and in the cytoplasm. The nuclear area was defined by the TO-PRO-3 signal (Supplementary Fig. 1d). PARP1 is a protein with both nuclear and cytoplasmic localisation and intensity values are presented without background subtraction. All measurements were performed using an in house-written programme for NIH-imageJ. Fluorescence images were obtained with either a Zeiss LSM 510-inverted confocal microscope or a Zeiss LSM 780-inverted confocal microscope, using a planapochromat 40X/NA 1.2 objectives. Through-focus maximum projection images were acquired from optical sections 0.5 μm apart and with a section thickness of 1.0 μm. H&E-stained images were obtained with a Leica scan system.

### Single-molecule RNA FISH (smFISH)

The probe targeting PARP1 was designed based on our previously described database covering all human transcripts (www.fusefish.eu) and consisted of 65 × 20 nt-long oligonucleotides targeting the transcript variant ENST00000366794 (Supplementary Table 1). We purchased oligos with a 3′-TEG amino modification from Biosearch Technologies, and coupled them to Alexa Fluor® 647 NHS Succinimidyl Ester (Molecular Probes, Cat. A20006) as previously described^29^. For smFISH, FFPE prostate biopsies were de-paraffinised in xylene, rehydrated, immersed 5 min in methanol-acetic acid 3:1 (v/v), and heated for 45 min at 80 °C in 0.01 M sodium citrate pH 6 supplemented with ribonucleoside vanadyl complex (RVC, NEB, Cat. S1402S) diluted 1:20 (v/v). All deparaffinisation steps were performed in special plastic jars (EMS, Cat. 71385) thoroughly decontaminated from RNases using RNaseZap® (Ambion, Cat. AM9780). All solutions were prepared in RNase-free water (Ambion, Cat. AM9939). After dehydration, 22 × 22 mm ‘Secure Seal’ hybridisation chambers (EMS, Cat. 70333-10) were mounted on each coverglass. Tissues were rehydrated and treated for 15 min with 0.025% pepsin in 0.01 M HCl. Auto-fluorescence was reduced by repeatedly flushing the chamber with freshly prepared 1% NaBH_4_ in 1X PBS solution, over a period of 15 min at room temperature. After washing in RNase-free water (3 times, 10 min each), the samples were washed in 2× SSC buffer (Ambion, Cat. 9763). Hybridisation, washings, mounting and imaging were performed as previously described^29^. Per field of view, we acquired 35 stacked images every 0.3 µm using a 100X magnification objective.

To quantify the smFISH signal, we selected the 5 most in-focus images in each stack and then counted all the mRNA molecules in each field of view using custom scripts in MATLAB^®^, as previously described^29^. To get an estimate of the mRNA density per cell (dots per µm^3^), we used an approach similar to what we recently described for quantifying HER2 and estrogen receptor transcriptional heterogeneity in breast cancer^30^: we split the z-projection of the mRNA dots identified in each stack into a regular grid of 13 × 13 squared pseudo-cells, and then divided the number of dots in each pseudo-cell by the number of focal planes minus one times the distance between each plane times the pseudo-cell area. In order to exclude possible background dots, we only considered pseudo-cells containing ≥3 mRNA dots.

### Reporter assay

In all luciferase assays, Renilla luciferase plasmid (Promega) was used as an internal control. All cells treated with androgens, R1881 (1 nM) were grown in hormone-depleted (charcoal-stripped) FBS. Cells were collected 48 h post-transfection using passive lysis buffer provided with dual luciferase assay reagents (Promega) to measure both luciferase activity and Renilla luciferase activity using luminometer (Pherastar).

### Cell viability assay

Cells were trypsinised and counted using a Vicell instrument. Cell growth assays were carried out in 96 well plates (1500–2000 cells per well). Cells were plated and simultaneously treated with the indicated chemicals/drugs until control (vehicle)-treated cells reached 95% confluence level (between 5 and 7 days for the different cell lines). Cell viability was determined by incubating the culture with MTS reagent (3-(4,5-dimethylthiazol-2-yl)-5-(3-carboxymethoxyphenyl)-2-(4-sulfophenyl)-2H-tetrazolium) followed by colorimetric assay as per manufacturers protocol (Promega).

### Live cell imaging/confluence analyses

Confluence analyses were performed using the Incucyte instrument (Essen Bioscience). Cells were plated and simultaneously treated with drugs in TPP 96-well culture plates and placed in a humidified chamber Incucyte instrument. Experiments were conducted with 8 replicates and live cell images were recorded every 3 h. For clarity of presentation, 12 h time interval data only are shown.

### Clonogenic assay

Cells were seeded in 6-well culture dishes (Corning); (1000 cells per well) and 48 h later were treated with antagonists (10 µM) and/or inhibitors (1 µM). Media and drugs were replenished bi-weekly. Two weeks later, cells were fixed in acetone:methanol (1:1) for 5 min and stained with Giemsa (Raymond A Lamb ltd.; diluted and filtered 1:10 in water) for 10–15 min. Plates were washed under running tap water, air dried and the colonies were counted using colony analyser (Oxford Optronix). All colonies were counted; experiments were conducted in triplicate.

### Immuno histo-chemistry for Ki67 and cleaved caspase-3 (CC3) of ex vivo culture

Immuno-histochemical staining of paraffin embedded slides was performed to detect Ki67 and CC3. Slides were stained on a BondMax Autostainer (Leica, Milton Keynes, UK). Antigen retrieval was performed using standard trisEDTA method at 100 °C for 20 min followed by a 15 min incubation with primary antibodies for Ki67 (Dako, M7240) and CC3 (cleaved caspase-3; 9664, Cell Signaling Technology) at room temperature, 8 min incubation with a secondary antibody (biotinylated donkey anti-rabbit; cat. No 711-065-152 from Jackson immunoresearch) using a polymer secondary system (Leica) followed by developing with DAB using enhancer (SP-2001; vector labs). Haematoxylin counterstaining was performed automatically on the Bond system, and finally, the slides were dehydrated, cleared and mounted using a Leica ST5020 attached coverslipper CV5030 (Leica). Slides were scanned onto Aperio/SpectrumTM v10.2.2.2317 and were analysed using ImageScope (Aperio software v12.0.0.5039). The intensity and number of nuclear Ki67 and CC3 was quantified using an algorithm that identifies nuclear staining, and the number of positive nuclei was counted. In order to identify epithelial structures for quantification, images, H&E- and cytokeratin-stained slides were used to pinpoint their exact location on serially sectioned slides. For each condition, at least 400 cells were counted and the percentage of positively stained cells was calculated.

### Neutral comet assay

Seventy-two hours after treatment with Doxycycline to induce shRNA for AR, C4-2 cells were exposed to IR and washed twice with PBS and collected by trypsinisation. Approximately 5 × 10^3^ cells in 10 μl of PBS (−) were mixed with 90 μl of LMAgarose (Trevigen), placed on GelBond Film (Lonza), covered with a 22 mm cover slide (VWR International) and left at 4 °C for 1 h. On removal of the cover slide, the cells were lysed with lysis solution (Trevigen) at 4 °C for 1 h. Following a wash with TBE (90 mM Tris borate (pH 8.3) and 2 mM EDTA), the samples were subjected to electrophoresis at 35 V, for 7 min in TBE. After washing with TBE, samples were fixed with 70% ethanol for 5 min at room temperature and dried overnight. The nuclei were stained with SYBR Green I (Invitrogen) in 10 mM Tris-HCl (pH 7.5) and 1 mM EDTA for 5 min at 4 °C. Images were taken with an IX71 fluorescent microscope (Olympus) with Cell^∧^F software (Olympus). Tail moments were measured using CometScore software (TriTek). The means of tail moment of at least 30–50 cells were measured per condition. Efficiency of DSB repair was determined as the tail moment ratio between the time points and the undamaged control cells obtained immediately after treatment.

### Generation of tumour xenografts

All experiments were carried out in compliance with the UK Animals (Scientifics Procedures) Act 1986 under a Project Licence and with the approval of the Cancer Research UK Cambridge Institute Animal Welfare and Ethical Review Body. Xenografts were generated in NSG mice by subcutaneous injection of cells. For PC3/PC3-AR xenografts, 1 million cells in 50 µl PBS were mixed with 50 µl Matrigel (BD Biosciences) and injected into the flanks region of each mouse (*n* = 6; both flanks). Once tumours (*n* = 8-12 per group) were palpable, mice were given vehicle (Cyclodextrin, Sigma Aldrich) or Olaparib (1 mg per mouse daily for 3 weeks) (LC laboratories) via the intra-peritoneal route. Tumour size was measured weekly using callipers. To generate C4-2 xenografts, 2 million cells in 50 µl PBS were mixed with 50 µl Matrigel and injected into the flank region of each mouse (*n* = 6 per group). Mice were given vehicle (DMSO) or Olaparib (50 mg/kg twice weekly) and/or bicalutamide (20 mg/kg twice weekly; Sigma-Aldrich) via the intra-peritoneal route. Tumour size was measured with callipers weekly. For all xenografts, tumour volumes were calculated using the formula volume = (π/6)/abc or (π/6)/abb (if only 2 diameters are available) and a,b,c are the orthogonal axis of the tumour. Mice were culled at completion of the experiment or when tumours reached 10% of body weight.

### Ex vivo prostate explant culture

Fresh human PCa tissue was collected after informed consent (70-year-old hormone-naive patient with cT3 disease with a PSA value of 82) and according to institutional policy. Excised tissue was cut into 1–2 mm^3^ size pieces and grown on collagen cushions placed on steel grids in RPMI with 10%FBS, 1% penicilln, streptomycin and gentamycin, and were treated with drugs for 1 week. Collagen cushions were prepared by solidification of 250 μl of collagen mix (rat tail collagen, plain RPMI media, FBS and 10% RPMI in the ratio of 7:1:1:1) on a nylon membrane. At the end of the experiment, the tissue was fixed in formalin for 20 h and then transferred to ethanol before paraffin embedding for immunohistochemistry.

### Statistical analyses

Descriptive statistics were reported as mean ± SEM for continuous variables and Student’s *t*-test was used where applicable. In case of more than two groups, ANOVA method was used. Appropriate nonparametric tests were used to analyse various datasets, which included Mann–Whitney U test and Holm–Sidak method. *P*-value of 0.05 or less was considered significant. *P < 0.05, **P < 0.01, ***P < 0.001.

### Data availability

All the other data that support the findings of this study are available from the corresponding authors upon reasonable request.

## Electronic supplementary material

Supplementary Information

Peer Review File
