# Supplementary material for: Synthetic lethality between androgen receptor signalling and the PARP pathway in prostate cancer
Source: Nat Commun. 2017 Aug 29;8:374. doi: 10.1038/s41467-017-00393-y (PMC5575038; doi:10.1038/s41467-017-00393-y)
Supplement: Supplementary file 1 — Supplementary Information [file 41467_2017_393_MOESM1_ESM.pdf]

File Name: Supplementary Information
Description: Supplementary Figures, Supplementary Table and Supplementary References

File Name: Peer Review File
Description:

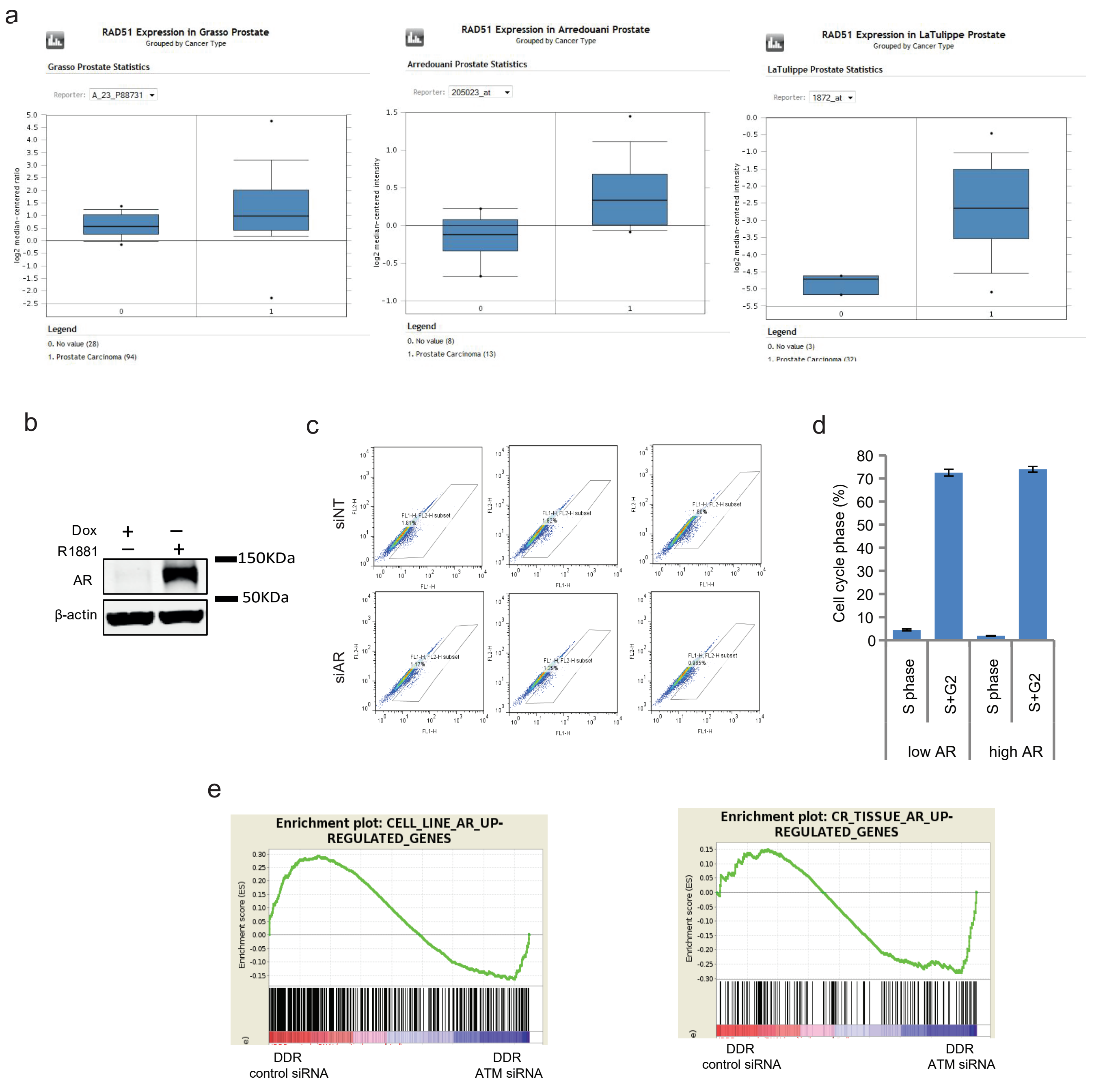

Supplementary Figure 1. Upregulation of HR factors in prostate cancer and link with AR signalling. (**a**) Distribution plot in Oncomine showing log2 expression value of RAD51 gene transcripts in three different PCa clinical gene expression data sets[1-3]. Plots show IQR, 95% CI and outlier points. (**b**) Western blot showing level of AR in “low AR” and “high AR” C4-2 cells treated with doxycycline (1 μg/ml) or androgen R1881 (1 nM) for 72 hr. (**c**) Flow cytometry plots for C4-2-DRGFP cells; boxes enclose cells which represent percentage of GFP positive cell population. (**d)** Flow cytometry data plotted as bar chart for cell cycle distribution(**e**) Gene set enrichment scores for DNA damage signatures for AR-regulated genes (AR occupied and down-regulated following castration in xenografts, GSE28126 and GSE21887).

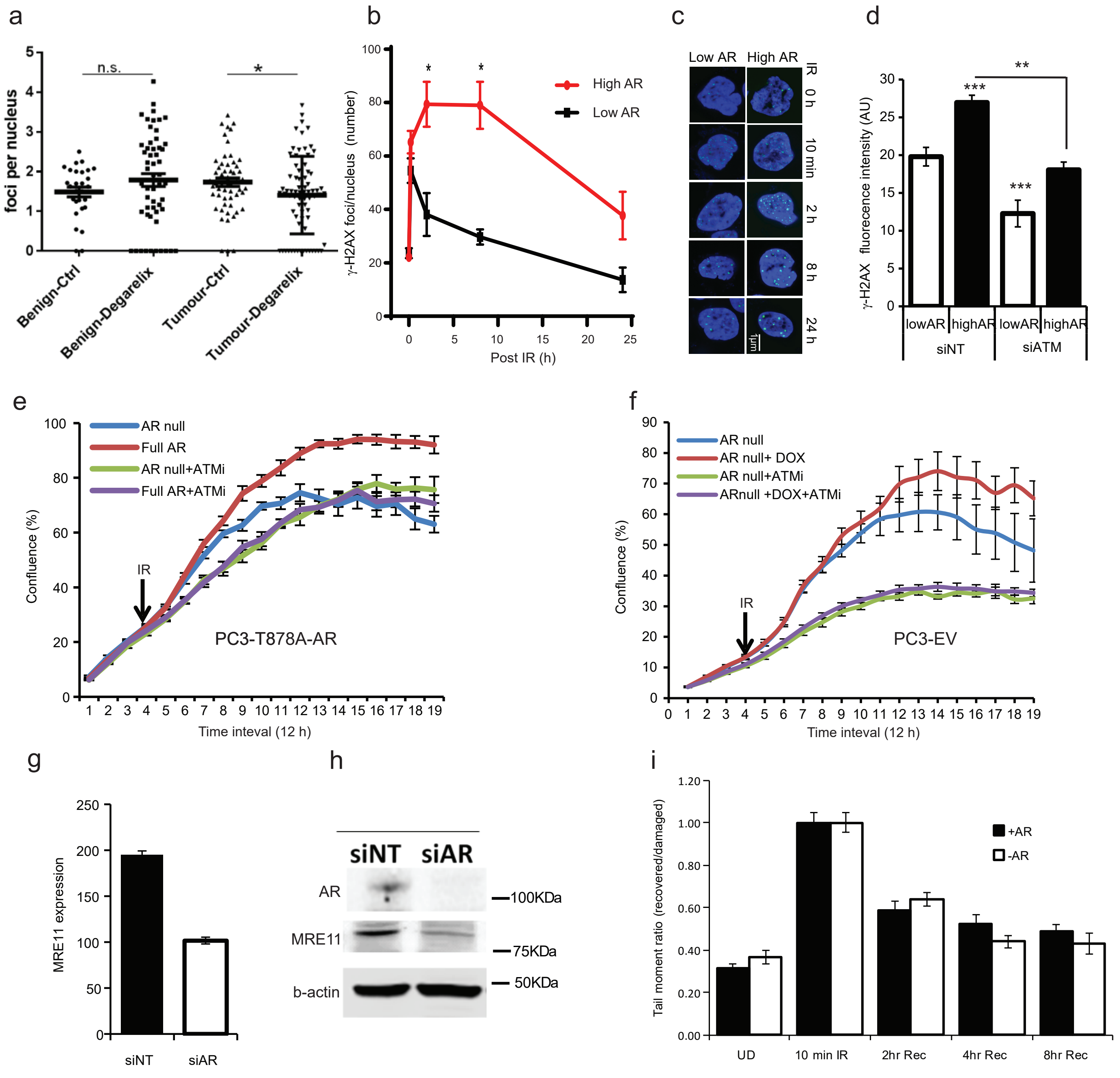

Supplementary Figure 2.Effect of AR signaling on DDR pathway in prostate cancer. (**a**) Scatter plot showing mean γH2AX foci per nucleus from benign glands and PCa tissue from patients treated with degarelix (number of patients=15) or tumour matched untreated control (number of patients=19). Each dot represents the average number of foci per nucleus in individual glands. Statistical significance was calculated by unpaired two-tailed Student's t-test ($p < 0.05$). (**b**) Foci analysis time course showing high content cytometry based quantification of the number of γH2AX foci in "low AR" and "high AR" C4-2 cell nuclei in response to radiation (10 Gy) at indicated time points. Statistical significance determined using the Holm-Sidak method, with alpha=5.000%. (**c**) Confocal microscopy images showing γH2AX foci (green dots) in the nuclei of "high AR" and "low AR" C4-2 cells at the indicated time after exposure to radiation. P value by two-sided Student's t-test. (**d**) High content cytometry analysis histogram show mean γH2AX levels per nucleus in "low AR" and "high AR" C4-2 cells transfected with siNT (non-targeting control) or siATM and measured 2 hr after exposure to radiation (10 Gy); data are presented as ± SEM. (**e**) Live cell imaging confluence analysis (Incucyte) of a dox-inducible mutant AR (T878A) expressing PC3 cell line (full AR) and its null counterpart (AR null) treated with ATMi (10 μM) for 2.5 days prior to and for 7 days following exposure to radiation (10 Gy as indicated by an arrow), standard error of mean is presented. (**f**) Live cell imaging confluence analysis (Incucyte) of AR null, PC3 empty vector cell line treated with doxycycline (1 μg/ml) and/or ATMi (10 μM) and exposed to radiation after 48 hr as indicated by arrow (10 Gy) for a total 156 hr; data represented as ±SEM. (**g**) Histogram showing MRE11 expression in LNCaP cells transfected with control siRNA (siNT) or siAR; data represented as ±SEM (**h**) Western blot of C4-2 cells transfected with control siRNA (siNT) or siAR, showing MRE11 protein expression under the stated conditions, with β-actin as the internal control. (**i**) Neutral comet assays in "high AR" and "low AR" cells.

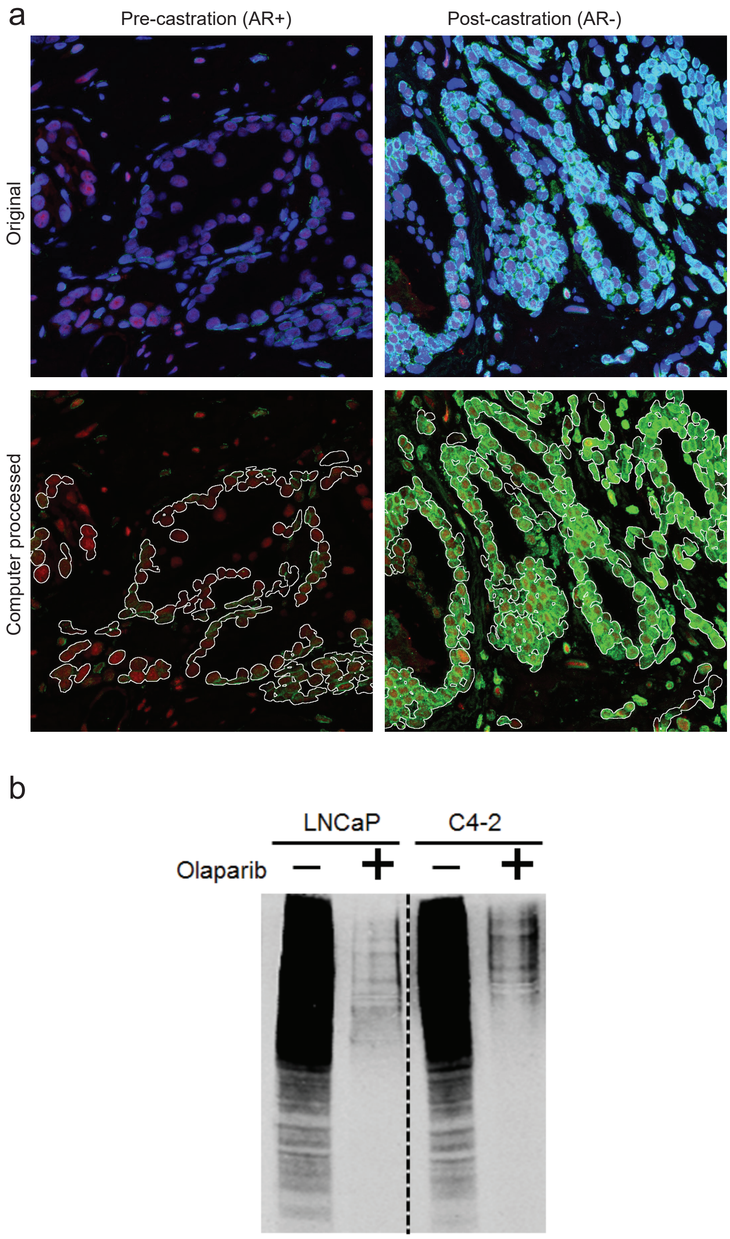

Supplementary Figure 3. PARP expression in prostate cancer cell lines and tissue.

(**a**) Immunofluorescence images showing original and computer processed images of the PCa tissue stained for PARP1 (red), PAR (green) and DNA (blue). White lines delineate the areas used for intensity measurement. (**b**) Western blot showing PARylated proteins from LNCaP and C4-2 cells treated with PARP-1/2 inhibitor Olaparib (1 μM) for 24 hr.

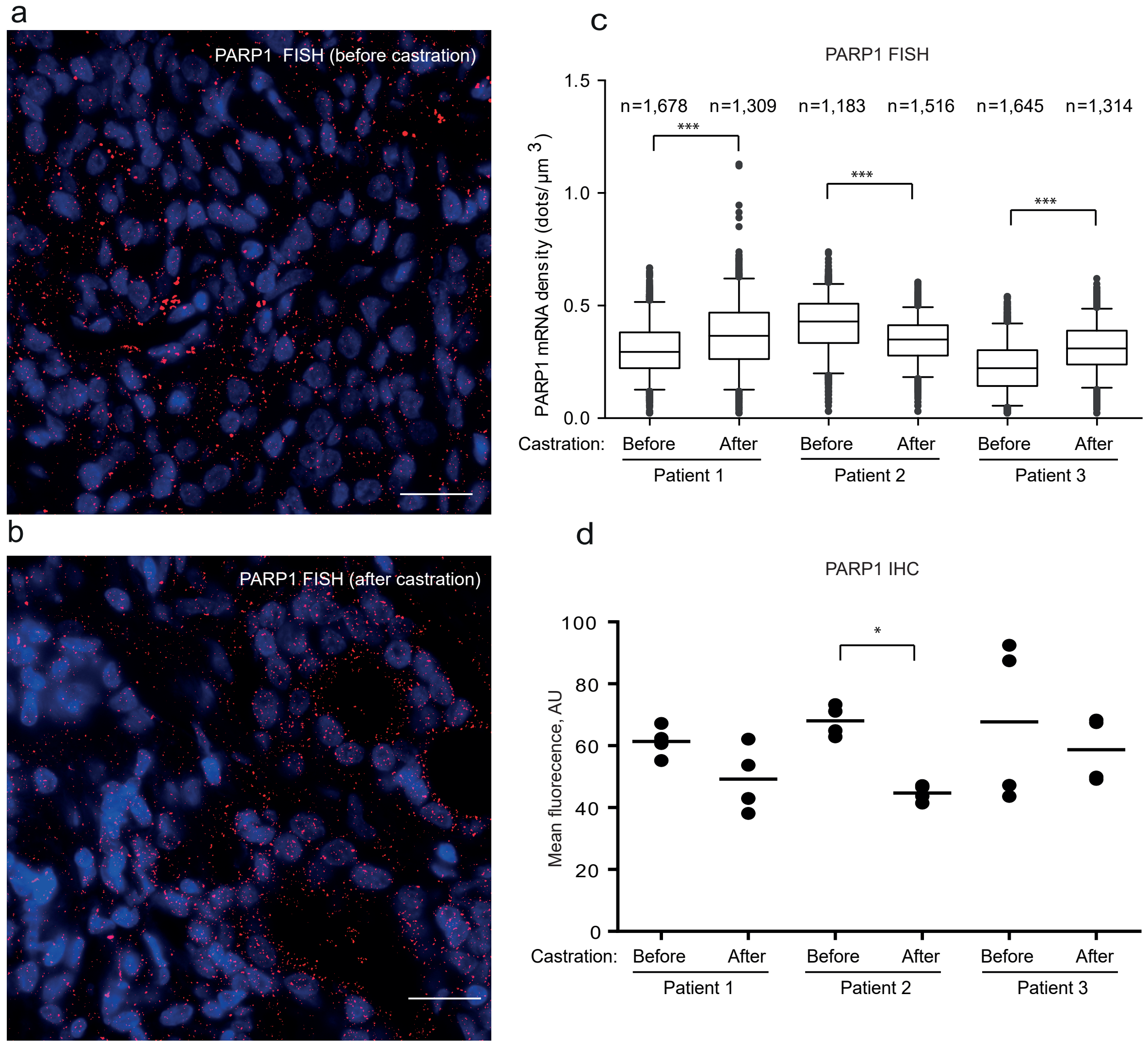

Supplementary Figure 4. Effect of castration on PARP1 mRNA levels. (**a-b**) PARP1 mRNA (red spots) expression in a fine-needle prostate biopsy taken from one patient before (**a**) and after (**b**) castration. The z-projection of the maximal intensity of 5 consecutive focal planes is shown. Scale bar, 20 μm. (**c**) Quantification of the density of PARP1 transcripts in samples derived from 3 patients before and after castration. Whiskers, 5–95 percentile range. Grey dots, outliers. *n*, number of pseudo-cells with ≥ 3 mRNA analyzed in each patient. *** = $p<0.001$ (Mann Whitney). (**d**) Effects of castration in PARP1 protein levels. The fluorescence of PARP1 antibody connected fluorophores has been quantified in the same patients and biopsies as in c. For details, see material and methods. * = $p<0.05$ (Mann Whitney).

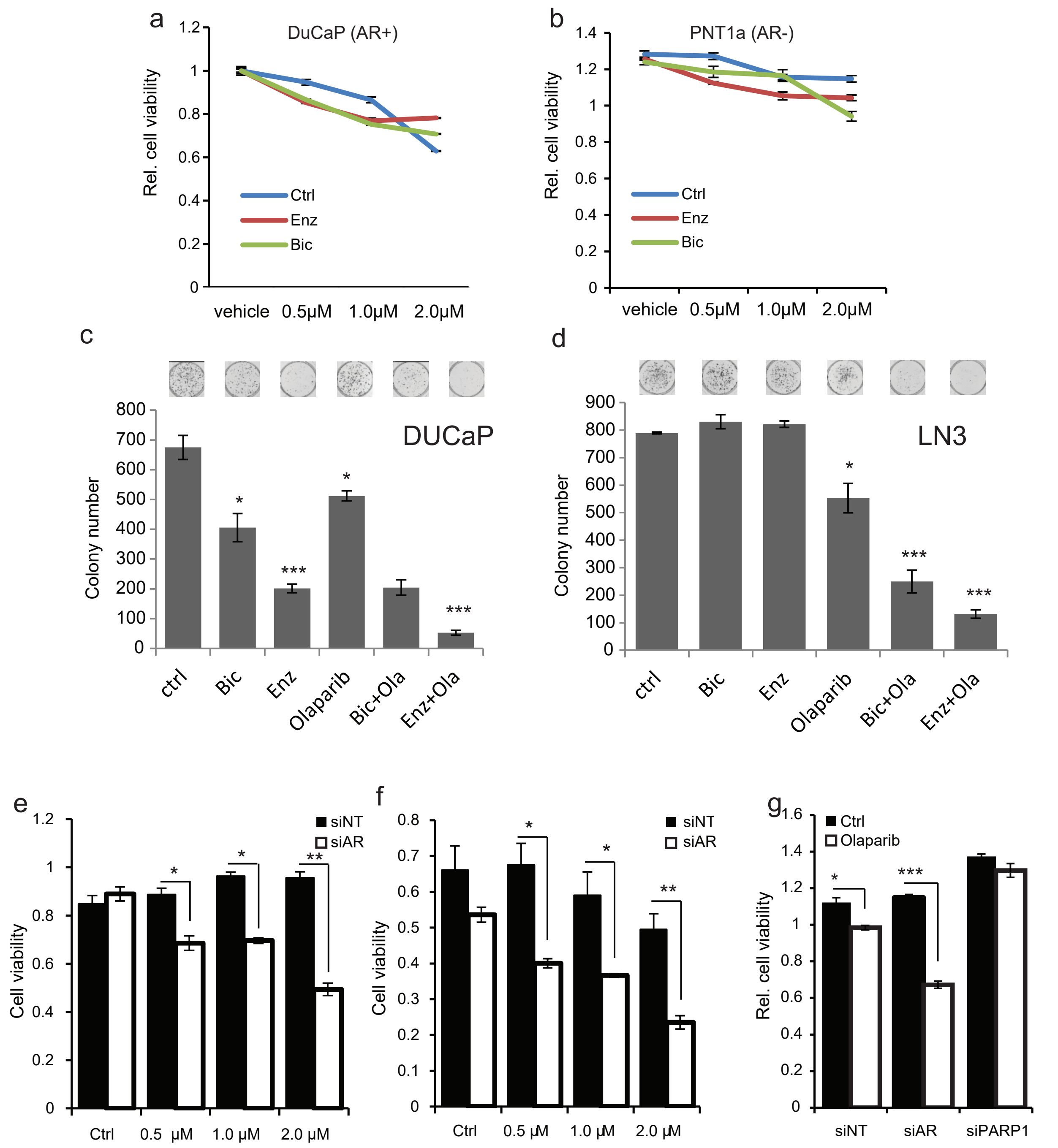

Supplementary Figure 5. Effect of PARP inhibitor and ADT on the growth of prostate cancer cell lines. (**a, b**) Viable fraction assessed by MTS assay of DUCaP (**a**) and (**b**) PNT1A cell lines treated with the indicated doses of Olaparib and/or enzalutamide or bicalutamide (10 μM) for 7 days or until 95% confluence was reached; bars show mean ±SEM. (**c** and **d**) Clonogenic cell survival assay for DuCAP (**d**) and LN3 (**e**) cells treated with bicalutamide or Enzalutamide (10 μM) and/or Olaparib (1 μM), P value by two-sided Student's t-test; bars show mean ±SEM. (**e** and **f**) Viable fraction of PC3-T878A AR (**e**) and C4-2 (**f**) cells transfected with control (siNT) and siAR and treated with the indicated doses of Olaparib for 5 days; P value by two-sided Student's t-test. (**g**) MTS assay results showing the viable fractions of C4-2 cells transfected with non-targeting control, AR or PARP1 siRNA and treated with Olaparib (1 μM) for 5 days; P value by two-sided Student's t-test.

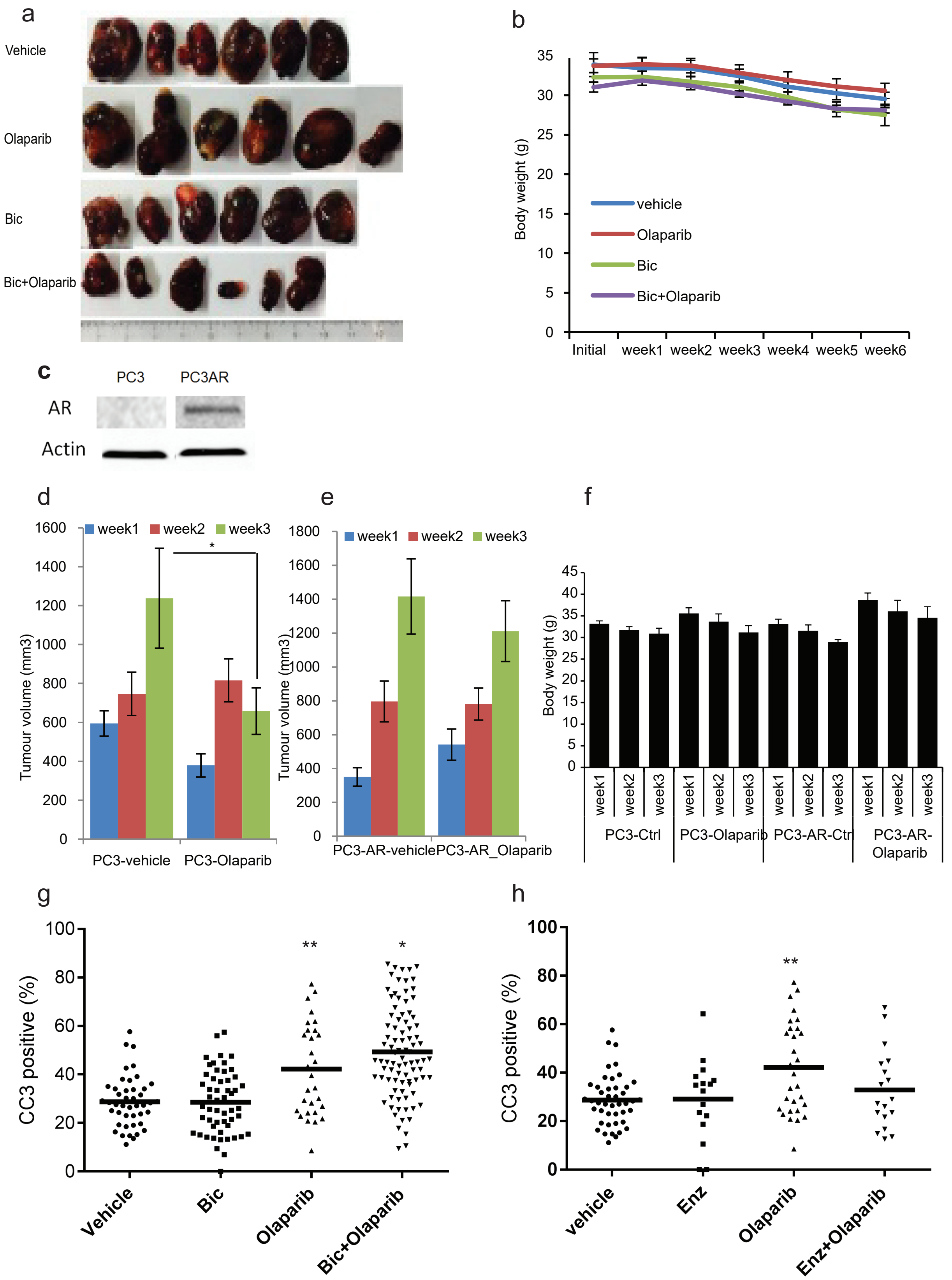

Supplementary Figure 6. Effect of PARP inhibitor and ADT on tumour xenograft and host animals. (**a**) Tumour images of C4-2 xenograft in NSG mice treated with indicated drugs. (**b**) Body weight of C4-2 xenograft bearing mice treated with the indicated drugs. (**c**) Western blot of PC3 and PC3-AR cells showing the AR protein expression in each cell line, β-actin is used as internal control. (**d** and **e**) Tumour xenografts of PC3 (d) or PC3-AR (e) cell lines, NSG mice were administered with DMSO (vehicle) or Olaparib as indicated, P value by two-sided Student's t-test. (**f**) Body weight of mice bearing PC3 or PC3-AR xenografts and treated with indicated drugs. (**g**) Quantification of cleaved caspase-3 expression in ex vivo culture of human PCa treated with bicalutamide (10 μM) and/or Olaparib (2 μM) for 72 hr; significance calculated by Mann-Whitney U test. (**h**) Quantification of cleaved caspase-3 expression in ex vivo culture of human PCa treated with Enzalutamide (10 μM) and/or Olaparib (2 μM) for 72 hr, significance calculated by Mann-Whitney U test.

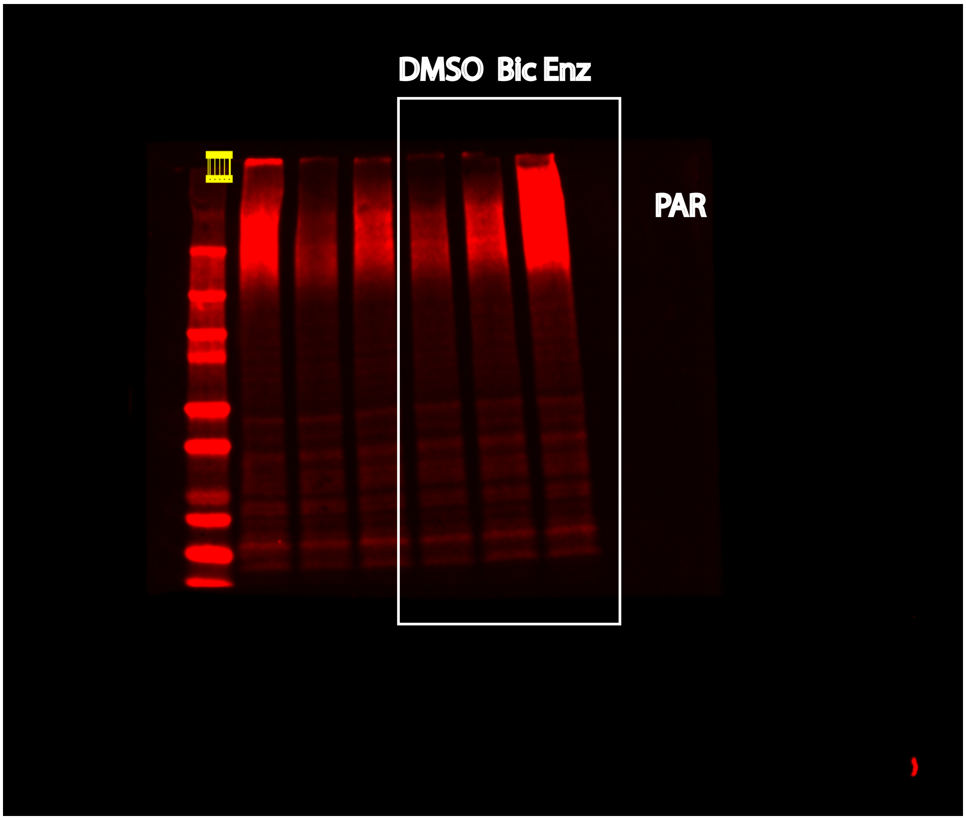

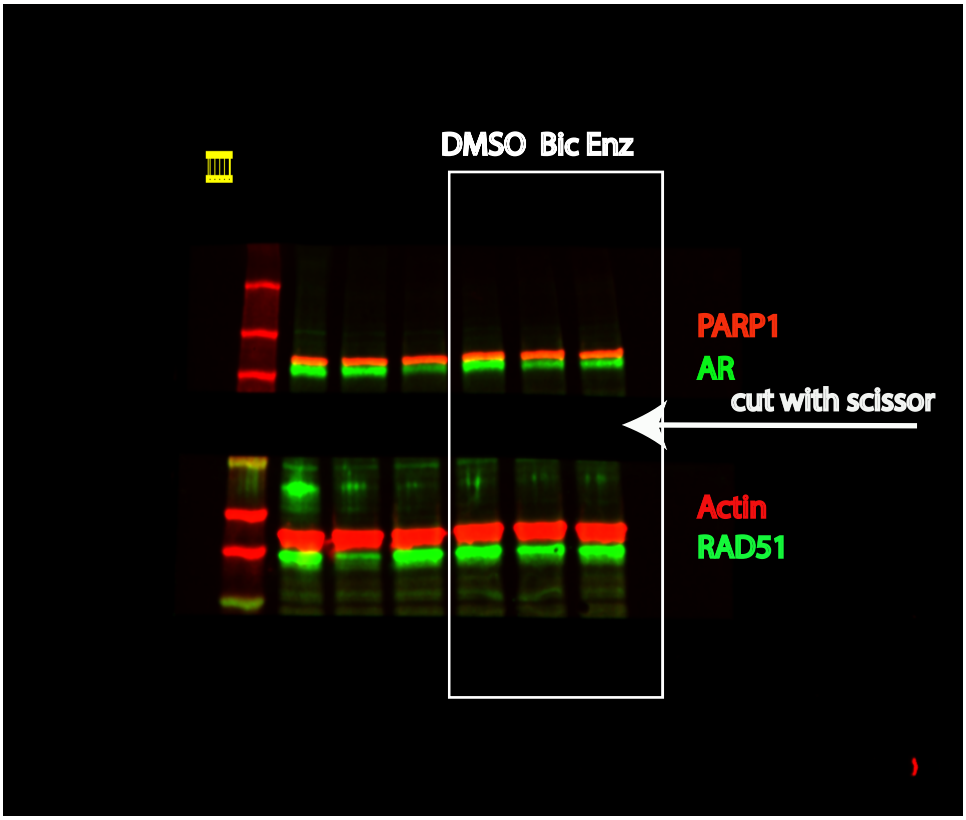

Supplementary Figure 7a: Uncropped Western blot image for figure 2a

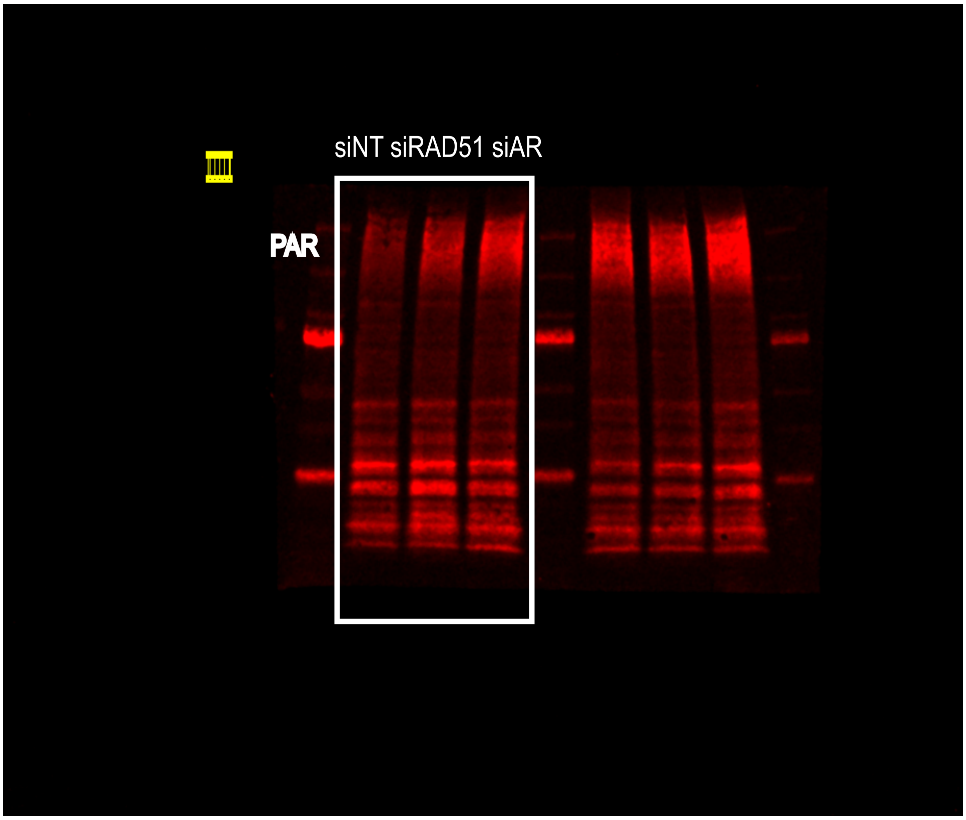

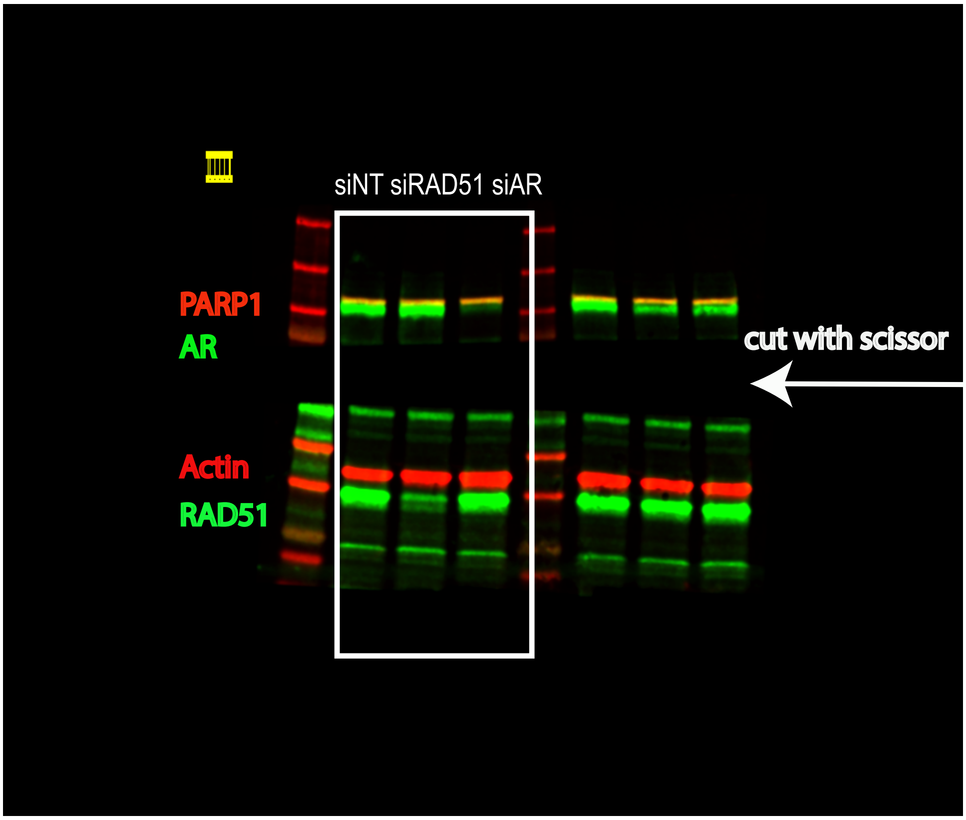

Supplementary Figure 7b: Uncropped Western blot image for figure 2b

Probe name	ENSEMBL gene	ENSEMBL transcript	Oligo #	Oligo sequence (5'->3')
PARP1	ENSG00000143799	ENST00000366794	PARP1_1	TTATCCGAAGACTCCGCCAT
PARP1	ENSG00000143799	ENST00000366794	PARP1_2	CTTGGCGTACTCGACTCGAT
PARP1	ENSG00000143799	ENST00000366794	PARP1_3	ACTGCACCATGATGGCCATC
PARP1	ENSG00000143799	ENST00000366794	PARP1_4	GGTACCAGTGTGGGACTTTT
PARP1	ENSG00000143799	ENST00000366794	PARP1_5	ACCTTCCAGAAGCAGGAGAA
PARP1	ENSG00000143799	ENST00000366794	PARP1_6	GAAGCTCAGAGAACCCATCC
PARP1	ENSG00000143799	ENST00000366794	PARP1_7	CAGCTTCCGCTGTCTTCTTG
PARP1	ENSG00000143799	ENST00000366794	PARP1_8	GAGTCTTCTCTGCCTTGCTA
PARP1	ENSG00000143799	ENST00000366794	PARP1_9	ACTTGGCATACTCTGCTGCA
PARP1	ENSG00000143799	ENST00000366794	PARP1_10	CCCCTTGCACGTACTTCTGT
PARP1	ENSG00000143799	ENST00000366794	PARP1_11	ATCTTCTTGGACAGGCGCAC
PARP1	ENSG00000143799	ENST00000366794	PARP1_12	TGGATGGTACCAGCGGTCAA
PARP1	ENSG00000143799	ENST00000366794	PARP1_13	CAGCTCCTCCCTGTTCTTGA
PARP1	ENSG00000143799	ENST00000366794	PARP1_14	AAGGAGGCTGAAGCCCTTGA
PARP1	ENSG00000143799	ENST00000366794	PARP1_15	AGATTTCTTCTTCGCCACTT
PARP1	ENSG00000143799	ENST00000366794	PARP1_16	GCCTTTAGGGCTTTTTCAAG
PARP1	ENSG00000143799	ENST00000366794	PARP1_17	GATGTTCCAGATCAGGTCGT
PARP1	ENSG00000143799	ENST00000366794	PARP1_18	ACACTTTCTTTAGCTCGTCC
PARP1	ENSG00000143799	ENST00000366794	PARP1_19	TGCTTGTTGAAGATGAGTAG
PARP1	ENSG00000143799	ENST00000366794	PARP1_20	ATGCCATCAGCTACTCGGTC
PARP1	ENSG00000143799	ENST00000366794	PARP1_21	TTGAAGACCAGCTGACCCGA
PARP1	ENSG00000143799	ENST00000366794	PARP1_22	CCCAGTGCAGTAATAGGCAT
PARP1	ENSG00000143799	ENST00000366794	PARP1_23	TGGGTGTCTGTGTCTTGACC
PARP1	ENSG00000143799	ENST00000366794	PARP1_24	TGAGGTAAGAGATTTCTCGG
PARP1	ENSG00000143799	ENST00000366794	PARP1_25	AGGAGTTCACAGCAGCAGGA
PARP1	ENSG00000143799	ENST00000366794	PARP1_26	GATAATGGCTTATCTGCTGA
PARP1	ENSG00000143799	ENST00000366794	PARP1_27	TCCTTGTTCCGGGACAGCTT
PARP1	ENSG00000143799	ENST00000366794	PARP1_28	AGTTTCTCAATCATGGCCTT
PARP1	ENSG00000143799	ENST00000366794	PARP1_29	TGCACAGGGAAGCCTTGTTG
PARP1	ENSG00000143799	ENST00000366794	PARP1_30	CCTCCATCTTCTTATTCATC
PARP1	ENSG00000143799	ENST00000366794	PARP1_31	CTCAGACACAACTCGGATGT
PARP1	ENSG00000143799	ENST00000366794	PARP1_32	TCCTGAAGGCTCTTGGTGGA
PARP1	ENSG00000143799	ENST00000366794	PARP1_33	GGACAAGATGTGCGCTAAGA
PARP1	ENSG00000143799	ENST00000366794	PARP1_34	TGGGGCCACAACTTCAACAG
PARP1	ENSG00000143799	ENST00000366794	PARP1_35	GTTGATACCTTCCTCCTTGA
PARP1	ENSG00000143799	ENST00000366794	PARP1_36	GAATCAGGATCCACAGCTGC
PARP1	ENSG00000143799	ENST00000366794	PARP1_37	GAAGACCTTCCCACCTTTCT
PARP1	ENSG00000143799	ENST00000366794	PARP1_38	TTGTAGTAGGAGTTGGTTCC
PARP1	ENSG00000143799	ENST00000366794	PARP1_39	AGGACCTGAATATCCAATAC
PARP1	ENSG00000143799	ENST00000366794	PARP1_40	GGACGGCATCTGTTCCAGTT
PARP1	ENSG00000143799	ENST00000366794	PARP1_41	GCGTTCCCGGTTTTTTCTTC
PARP1	ENSG00000143799	ENST00000366794	PARP1_42	GGGGGTAGAACTTTTTGGGA
PARP1	ENSG00000143799	ENST00000366794	PARP1_43	CATCCTGGCCATAGTCAATC
PARP1	ENSG00000143799	ENST00000366794	PARP1_44	CAAAGATCATCTTGATGAGG
PARP1	ENSG00000143799	ENST00000366794	PARP1_45	CTGAAGGTCGATCTCATACT
PARP1	ENSG00000143799	ENST00000366794	PARP1_46	ATGGAGTATGCGGCCTGGAT
PARP1	ENSG00000143799	ENST00000366794	PARP1_47	AGGATCTGAGAGTCGCTGCT
PARP1	ENSG00000143799	ENST00000366794	PARP1_48	AGGGTGTAAAAGCGATTTGA
PARP1	ENSG00000143799	ENST00000366794	PARP1_49	TTGTTCAGGAGCGGAGGCTT
PARP1	ENSG00000143799	ENST00000366794	PARP1_50	CTCGATGTCCAGCAGGTTGT
PARP1	ENSG00000143799	ENST00000366794	PARP1_51	GTTGACATCGATGGGATCCT
PARP1	ENSG00000143799	ENST00000366794	PARP1_52	GGCTTCTTCAGAATCTCTGT
PARP1	ENSG00000143799	ENST00000366794	PARP1_53	CCAAGTCATACGCATTGTGT
PARP1	ENSG00000143799	ENST00000366794	PARP1_54	AACGCTGGCATTCGCCTTCA
PARP1	ENSG00000143799	ENST00000366794	PARP1_55	ATGAAGCTGCTTAAAGGGCT
PARP1	ENSG00000143799	ENST00000366794	PARP1_56	GATCCCAGCAAAGTTGGTGG
PARP1	ENSG00000143799	ENST00000366794	PARP1_57	TACCAAACATGTAGCCTGTC
PARP1	ENSG00000143799	ENST00000366794	PARP1_58	GTTGGCACTCTTGGAGACCA
PARP1	ENSG00000143799	ENST00000366794	PARP1_59	GCAACTTCTCCCAACAGGAT
PARP1	ENSG00000143799	ENST00000366794	PARP1_60	TGGGTAACTTGCTGATATGT
PARP1	ENSG00000143799	ENST00000366794	PARP1_61	CAGGGGTAGTTTTGCCCAAA
PARP1	ENSG00000143799	ENST00000366794	PARP1_62	CAAGAGGAACGTCTACACCA
PARP1	ENSG00000143799	ENST00000366794	PARP1_63	CGTTATATAGTAGAGAGGTG
PARP1	ENSG00000143799	ENST00000366794	PARP1_64	CCTGAGCAATATCATAGACA
PARP1	ENSG00000143799	ENST00000366794	PARP1_65	CAGGGAGGTCTTAAAATTGA

Supplementary Table 1. List of oligo sequences used as smFISH probe targeting PARP1

Supplementary References

1. Grasso, C.S., *et al.* The mutational landscape of lethal castration-resistant prostate cancer. *Nature* **487**, 239-243 (2012).
2. Arredouani, M.S., *et al.* Identification of the transcription factor single-minded homologue 2 as a potential biomarker and immunotherapy target in prostate cancer. *Clin Cancer Res* **15**, 5794-5802 (2009).
3. LaTulippe, E., *et al.* Comprehensive gene expression analysis of prostate cancer reveals distinct transcriptional programs associated with metastatic disease. *Cancer Res* **62**, 4499-4506 (2002).
