# Supplementary material for: Synthetic lethality between androgen receptor signalling and the PARP pathway in prostate cancer
Source: Nat Commun. 2017 Aug 29;8:374. doi: 10.1038/s41467-017-00393-y (PMC5575038; doi:10.1038/s41467-017-00393-y)
Supplement: Supplementary file 2 — Peer Review File [file 41467_2017_393_MOESM2_ESM.pdf]

Editorial Note: this manuscript has been previously reviewed at another journal that is not operating a transparent peer review scheme. This document only contains reviewer comments and rebuttal letters for versions considered at Nature Communications.
